# The predictive effect of immune therapy and chemotherapy under T cell-related gene prognostic index for Gastric cancer

**DOI:** 10.3389/fcell.2023.1161778

**Published:** 2023-05-18

**Authors:** Jingyao Chen, Xing Li, Tsz Kin Mak, Xiaoqun Wang, Hui Ren, Kang Wang, Zi Chong Kuo, Wenhui Wu, Mingzhe Li, Tengfei Hao, Changhua Zhang, Yulong He

**Affiliations:** ^1^ Digestive Diseases Center, The Seventh Affiliated Hospital of Sun Yat-sen University, Shenzhen, China; ^2^ Guangdong Provincial Key Laboratory of Digestive Cancer Research, The Seventh Affiliated Hospital of Sun Yat-sen University, Shenzhen, Guangdong, China; ^3^ Department of Gastrointestinal Surgery, The First Affiliated Hospital of Sun Yat-sen University, Guangzhou, Guangdong, China

**Keywords:** T-cell score, T Cell related gene, gastric cancer, immune therapy, immune microenvironment infiltration

## Abstract

**Background:** Gastric cancer (GC) is one of the most common malignancies in the human digestive tract. CD4+T cells can eliminate tumor cells directly through the mechanism of cytolysis, they can also indirectly attack tumor cells by regulating the tumor TME. A prognostic model of CD4+T cells is urgently needed to improve treatment strategies and explore the specifics of this interaction between CD4+T cells and gastric cancer cells. Methods: The detailed data of GC samples were downloaded from the Cancer Genome Atlas (TCGA), GSE66229, and GSE84437 datasets. CD4^+^ T cell-related genes were identified to construct a risk-score model by using the Cox regression method and validated with the Gene Expression Omnibus (GEO) dataset. In addition, postoperative pathological tissues of 139 gastric cancer patients were randomly selected for immunohistochemical staining, and their prognostic information were collected for external verification. Immune and molecular characteristics of these samples and their predictive efficacy in immunotherapy and chemotherapy were analysed.

**Results:** The training set and validation set had consistent results, with GC patients of high PROC and SERPINE1 expression having poorer prognosis. In order to improve their clinical application value, we constructed a risk scoring model and established a high-precision nomogram. Low-risk patients had a better overall survival (OS) than high-risk patients, consistent with the results from the GEO cohort. Furthermore, the risk-score model can predict infiltration of immune cells in the tumor microenvironment of GC, as well as the response of immunotherapy. Correlations between the abundance of immune cells with PROC and SERPINE1 genes were shown in the prognostic model according to the training cohort. Finally, sensitive drugs were identified for patients in different risk subgroup.

**Conclusion:** The risk model not only provides a basis for better prognosis in GC patients, but also is a potential prognostic indicator to distinguish the molecular and immune characteristics of the tumor, and its response to immune checkpoint inhibitor (ICI) therapy and chemotherapy.

## Introduction

Gastric cancer (GC) is one of the most common malignancies in the human digestive tract. According to the global cancer statistical analysis data, GC has become the sixth most diagnosed cancer and the third largest cause of cancer death, and thus it is a major global health crisis (Sung et al., 2021). Clinically, GC is mainly treated with surgical resection, chemotherapy, radiotherapy, targeted therapy, or in combination. In spite of this, patients still face many life-threatening issues like recurrence, metastasis, drug resistance, lack of corresponding drug targets, side effects and so on. The 5-year survival rate is as low as 10%–15% (Li Y. et al., 2020; Smyth et al., 2020), hence exploring new and more effective treatment methods have become a hot spot of research.

Immunotherapy is based on the study of the mechanism of immune escape, which can reactivate the anti-tumor immune response and overcome the escape pathway by “manipulating” the immune system (Kennedy and Salama, 2020). In recent years, it has been used to treat malignancies as a new treatment model, and has shown good therapeutic effects. Tumor microenvironment (TME), due to its key role in cancer progression and drug resistance, has become a potential immunotherapy target for many kinds of malignant tumors, including GC (Rihawi et al., 2021).

TME consists of different types of cells, including immune and inflammatory cells (lymphocytes and macrophages), stromal cells (fibroblasts, adipocytes and pericytes), small organelles, RNA, blood and lymphatic vessels, extracellular matrix (extra cellular matrix, ECM) and secreted proteins (Arneth, 2019). Many studies have reported that the occurrence and development of any tumor and the clinical prognosis of patients are closely related to the level of infiltration of tumor immune cells (Hainaut and Plymoth, 2013; Dashti et al., 2016; Kono et al., 2020). As a member of the immune regulatory network, the changes in immune-related genes (IRGs) will cause cascade reactions, thus promoting the progression of tumors (Zhang S. et al., 2020). Studies have shown that IRGs are closely related to the occurrence, development and metastasis of tumors. For example, studies have shown that the overexpression of YKT6 is closely related to the poor prognosis of OSCC, and its low expression is associated with the high level of CD8+T cells in OSCC and the potential response to immunotherapy (Yang et al., 2021). The high expression of HCST is closely related to the level of tumor infiltrating immune cells, especially dendritic cells, and is closely related to the clinicopathology and poor prognosis in renal clear cell carcinoma as well (Zhou et al., 2021). Gene ANGPT1 can increase T Cell infiltration and improve the prognosis of EC patients (Nong et al., 2021). CD4+T cells are a special type of T cells that can target tumor cells in many ways. On the one hand, they can eliminate tumor cells directly through the mechanism of cytolysis, while on the other, they can indirectly attack tumor cells by regulating the tumor TME (Kennedy and Celis, 2008; Melssen and Slingluff, 2017). Additionally, CD4^+^ T cells can also kill tumor cells by increasing the number and quality of B Cells and CTL (Cytotoxic T Lymphocytes) responses (Bevan, 2004; Castellino and Germain, 2006).

Lately, the emergence of immune checkpoint inhibitor (ICI) represented by PD-1 (programmed cell death protein-1) has brought about a new dawn of treatment for tumor patients. Tumor tissue disables our T cells by expressing programmed death molecules such as PD-1 and B7-1 and subsequently binding them to PD-L1 (programmed death ligand-1) and CTLA-4 (cytotoxic T lymphocyte antigen-4) receptors on said T cells (Sharma and Allison, 2015). By blocking this binding, ICI keeps T cells alert and capable of searching and destroying tumor cells (Ganesh et al., 2019). ICI therapy is effective in the treatment of melanoma and non-small cell lung cancer, for example, and can be as effective as 50% in advanced melanomas (Mushti et al., 2018; Carlino et al., 2021). Hence, seeking for more potential targets for immunotherapy is understandably prioritized in many bleeding-edge studies. At present, there are many studies on immunotherapy for gastric cancer, but most of these studies only focus on one or two genetic biomarkers related to the prognosis of gastric cancer, which is far from enough. At the same time, more evidences are required to detect specific characteristics in GC patients which makes immunotherapy effective.

Therefore, in this study, WGCNA (weighted gene co-expression network analysis) was used to construct a network map of co-expression of immune cells, from which the key marker related to gastric cancer was screened. Then the risk-score model was established and verified in clinical practice in the aspects of prognosis, immune microenvironment and drug sensitivity of patients with gastric cancer. Thus, it provides important insights and strategies for individualized treatment of patients with gastric cancer.

## Methods

### Datasets collection of GC and preprocessing

The flow chart (Supplementary Figure S1) shows sample utilization at each stage of the statistical analysis. Data such as somatic mutation, gene expression, and corresponding clinical information of gastric cancer (GC) samples were collected for further analysis from The Cancer Genome Atlas (TCGA) database (https://tcga-data.nci.nih.gov/tcga/), which includes detailed information of tumor and para tumor samples. In addition, detailed characteristics and survival time of 433 gastric cancer samples in South Korea (GSE84437) and 300 gastric cancer samples in the ACRG (Asian Cancer Research Group) study (GSE66229) were obtained from the GEO database (https://www.ncbi.nlm.nih.gov/geo/).

### Evaluation of immune cell infiltration

CIBERSORT is used to dissect the mixture of data from unknown content and noise. The algorithm can statistically estimate the relative proportions of subtype populations in complex tissue expression profiles and is a useful tool for estimating specific cell abundances in mixed tissues. We used CIBERSORT algorithm to analyze RNA-seq data of GC patients to estimate the relative proportions of various immune-infiltrating cells and its content. Then, each subtype of immune cells in these GC sample was used as trait data for WGCNA.

### Construction of co-expression network

In this study, we have selected 493 genes to construct a weight co-expression network in order to identify the relationship between functional modules and immune cell infiltration in GC patients. According to the Pearson correlation value between paired genes, the expression levels of individual transcripts were transformed into a similarity matrix, and then to an adjacency matrix, as calculated by amn = |cmn| *β* (cmn = Pearson’s correlation between paired genes; amn = adjacency between paired genes). Parameter *β* can enhance the strong correlation between genes and decrease the weak correlation. When the power of *β* is set to 6, the adjacency matrix was converted into a topological overlap matrix. To divide genes with similar expression patterns into different modules, we applied a dynamic hybrid cutting method by using a bottom-up algorithm with a minimum block size truncation of 60.

### Identification of hub genes

The candidate hub genes were selected according to the module connectivity and immune cell relationship of each gene in hub module. Module connectivity is defined as the absolute value of the Pearson’s correlation between genes (Module Membership). The immune cell relevance is defined as the absolute value of the Pearson’s correlation between each gene and its trait (Gene Significance). For each gene, we define the MM by the correlation between the gene expression profile and the ME (Module Eigengenes) of a given module. For example, MMturquoise (a) = cor (xa, Eturquoise) measures the correlation between gene “a” and the ME of the turquoise module. If MMturquoise (a) is close to 1 or −1, it is highly connected to the ME of the turquoise module. On the other hand, if MMturquoise (a) is close to 0, the “a” gene is not part of the turquoise module. In this study, we selected the MEblue module that is highly relevant to activated memory CD4^+^ T cells for further analysis.

### Function enrichment analyses

To verify the biological functions of modules, we employed the gene ontology (GO) annotation and Kyoto encyclopedia of genes and genomes (KEGG) pathway enrichment analysis by clusterProfiler R package (Huang da et al., 2009; Yu et al., 2012). The parameters of clusterProfiler R package were set to default. The thresholds of the GO functions and KEGG pathways were set as *p*-value <0.05 and qvalue <0.05 respectively. Gene Set Enrichment Analysis (GSEA) is used to identify a set of basically defined genes which exhibit statistical differences between two biological states (Hänzelmann et al., 2013). “c2.cp.kegg.v7.4.symbols.gmt” gene set enrichment analysis was executed according to gene expression, with *p*-value <0.05 and q-value <0.05 as indicative of statistical significance. In this study, we applied GSEA to find the signaling pathways of core genes by R packages “ggplot2” and “clusterProfiler”.

### Construction and validation of prognostic model

In our research, we used Lasso-Cox analysis to minimize the risk of over-fitting by using the “glmnet” R package. Multivariate Cox analysis was used to select the candidate genes for establishing a prognostic risk-score in the training cohort. The risk-score was calculated as follows:

$$Risk-score=\Sigma i\left(Expi\;*\;coefi\right)$$

where Coefi and Expi denote the risk coefficient and expression of each gene, respectively. The cut-off point was determined by the “survminer” package. According to the risk-score, we showed that the survival curve was used for visualization with both training and testing cohorts in the high- or low-risk group by Kaplan-Meier analysis. *p* values <0.05 were considered to be statistically significant.

### *In vitro* validation and survival analyses

Surgically treated and pathologically confirmed GC patients (*n* = 139) between 2010 and 2012 from the First Affiliated Hospital of Sun Yat-sen University (FAHSYSU) were randomly selected. The follow-up period was up to January 2019.139 paraffin-embedded GC specimens were obtained from Department of Pathology of FAHSYSU, and their IHC staining and tissue microarrays were conducted using an anti-PROC antibody (1:200; Proteintech, Wuhan, China) and anti-SERPINE1 antibody (1:100; Proteintech, Wuhan, China) as previously described. IHC results were evaluated by two independent investigators blinded to the experiments, and a semiquantitative method was used to score the specimens (Zhai et al., 2018). Positive was defined as samples in which more than 10% of the tumor cells were stained. The staining intensity was defined as follows: 0 (negative), 1 (weak), 2 (moderate), and 3 (strong). Negative to weak staining indicated low PROC and SERPINE1 expression, and moderate to strong staining indicated high PROC and SERPINE1 expression. Patient consent and ethical approval from the Institutional Review Board of Seventh Affiliated Hospital of Sun Yat-sen University were obtained for this study. Statistical analyses were performed using SPSS 22.0. The chi-square test was used for numerical data. Survival curves were generated using the Graphpad Prism 8.0.

### Assessment of immunotherapy

In further analysis, we showed the correlations between the abundance of immune cells and two genes, specifically PROC and SERPINE1 in the prognostic model according to the training cohort. Beside comparing the prognostic among the risk-score, we also utilized the immunophenoscore (IPS) to predict the response of immune checkpoint inhibitors (ICIs) based on the expression of the main component in tumor immunity. On a scale of 0–10 based on representative z-scores of cell type gene expression, IPS was calculated where the immunogenicity was positively correlated to its IPS (Charoentong et al., 2017). The IPSs of patients with GC were derived from The Cancer Immunome Atlas (TCIA) (http://tcia.at/home). The result was obtained using the R package “ggpubr”.

### Establishment and validation of a nomogram scoring system

According to the independent prognosis outcome, a predictive nomogram was created by incorporating clinical characteristics and risk-score using the R package “rms”. In the nomogram scoring system, each variable has a corresponding score and the total score is obtained by adding up the scores of all variables for each sample (Iasonos et al., 2008). The Nomogram was evaluated using ROC curves for 1-, 3-, and 5-year survival rates. The nomogram calibration plots were used to describe the predictive value of the anticipated 1-, 3-, and 5-year survival events in relation to the actual observed outcomes.

### Assessment of drug sensitivity

The sensitivity of various drugs was predicted between high-risk and low-risk subgroups using the R package “pRRophetic” (Geeleher et al., 2014). The differences in IC50 between the two groups was compared using Wilcoxon signed-rank test. The results were plotted using the R package “ggplot2”.

### Statistical analysis

We used R software for statistical analyses (version 4.1.3; https://www. R-project.org). The significance of different immune cell infiltration and gene expression was calculated using Wilcoxon test analysis. Pearson correlation analysis was used to calculate the correlation between genes (Module Membership). When the *p* < 0.05, the result was considered statistically significant.

## Results

### Differentially expressed immune-related genes and analysis of immune cell infiltration in GC

**FIGURE 1:**
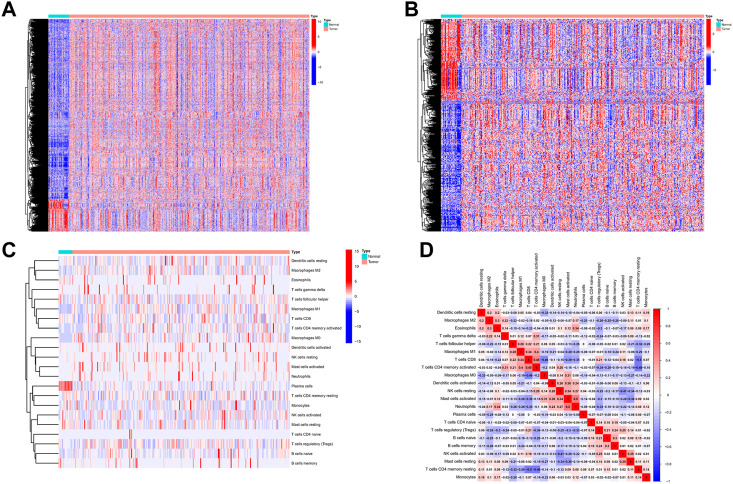
Differentially expressed immune-related genes and Analysis of Immune Cell Infiltration in GC. **(A)** Differentially expressed immune-related genes from the intersecting list. **(B)** Differentially expressed genes retrieved from the TCGA cohort. **(C)** The heat map of the immune cells between tumor and normal. **(D)** Correlation analysis of immune cells.

The process flow chart of our study was clearly illustrated in Supplementary Figure S1. Initially, we obtained the transcriptome profiling data of Stomach adenocarcinoma (STAD) project from the Cancer Genome Atlas (TCGA) database, which included 375 tumor samples and 32 normal samples. Through differential expression genes analysis, there were 8,832 differentially expressed genes retrieved from the TCGA cohort, including 7,497 upregulated genes and 1,335 downregulated genes in the tumor samples when compared with normal samples (Supplementary Table S1A) (Figure 1A). Meanwhile, we cross-referenced these differential genes with the lists of immune-related genes retrieved from ImmPort and InnateDB. A total of 493 differentially expressed immune-related genes were obtained from the list, which can be broken down into 309 upregulated genes and 184 downregulated genes in the tumor samples when compared with normal samples (Supplementary Table S1B) (Figure 1B). In addition, we analyzed the different cell subtype abundance between GC and normal tissues by using the CIBERSORT algorithm. As a result, we identified the composition of immune cells in each GC sample (Supplementary Figure S2). The immune cells between tumor and normal sample are presented in the heat map (Figure 1C). The correlation analysis of immune cells can be found in Figure 1D. These results suggested that the aforementioned immune cells may be involved in the progression of GC.

### Construction weighted gene Co-Expression network

**FIGURE 2:**
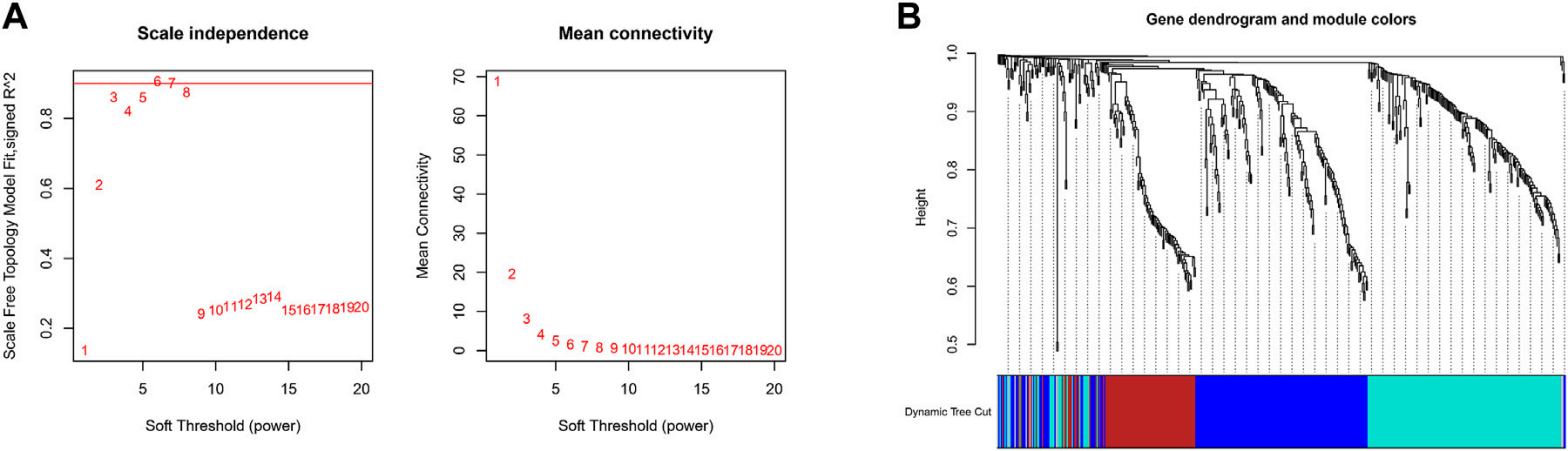
Construction Weighted Gene Co-Expression Network. **(A)** The scale-free fit index of the network topology in the WGCNA pipeline. **(B)** Dynamic hybrid cutting to construct hierarchical clustering tree.

The expression values of these 493 genes were utilized to build a co-expression network of GC using the R package “WGCNA”. We clustered the samples of TCGA by estimating average linkage and Pearson’s relation values. Soft threshold power analysis revealed the scale-free fit index of the network topology in the WGCNA pipeline. We used *β* = 6 as the soft thresholding value to build a scale-free network (Figure 2A). Dynamic hybrid cut was utilized to construct hierarchical clustering tree. Each leaf of the tree shows a single separate gene, in which genes with similar expression data are grouped close together to form a branch of the tree representing a gene module (Figure 2B).

### Identification of hub modules and validation of enrichment analysis

**FIGURE 3:**
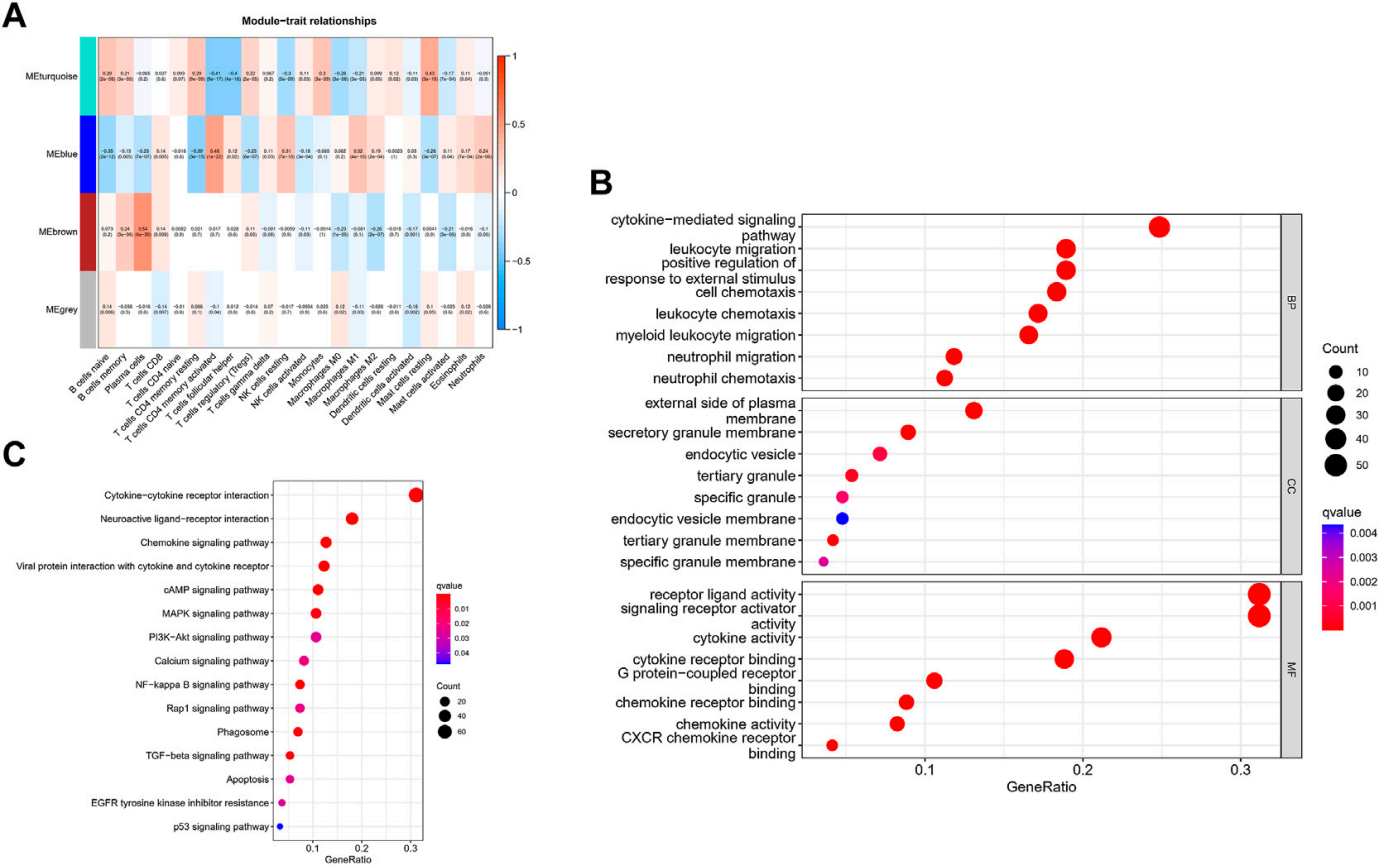
Identification of hub modules and Validation of enrichment analysis. **(A)** The correlation between the modules and immune cell. **(B)** The GO enrichment analysis of the MEblue module. **(C)** The ICEGG pathway enrichment analysis of the MEblue module.

The correlation between the modules and immune cell is showed in Figure 3A. Compared with other immune cells, the relationship between activated memory CD4^+^ T cells and GC is less studied. In this study, the MEblue module was highly related to activated memory CD4^+^ T cells. All genes in the MEblue module are shown in Supplementary Table S2. Hence, we selected MEblue module and activated memory CD4^+^ T cells for further analysis. There was a very significant correlation between module membership and gene significance (cor = 0.48, p = 1E-22), suggesting that the 173 genes in the MEblue module tended to be significantly correlated with the infiltration level of activated memory CD4^+^ T cells. For this reason, the MEblue module was considered to be a GC-related hub module. To illustrate the affected functions of the genes clustered in the MEblue module, GO and KEGG analysis was further performed. Based on the GO enrichment analysis, cellular response to biotic stimulus, cellular response to molecule of bacterial origin, response to lipopolysaccharide, neutrophil chemotaxis, and neutrophil migration were tagged as significantly enriched GO terms (Figure 3B). The KEGG pathway enrichment analysis revealed that most genes were mainly enriched in pathways including leukocyte chemotaxis, myeloid leukocyte migration, cell chemotaxis, and so on. (Figure 3C).

### Establishing risk assessment model and survival outcomes in GC

**FIGURE 4:**
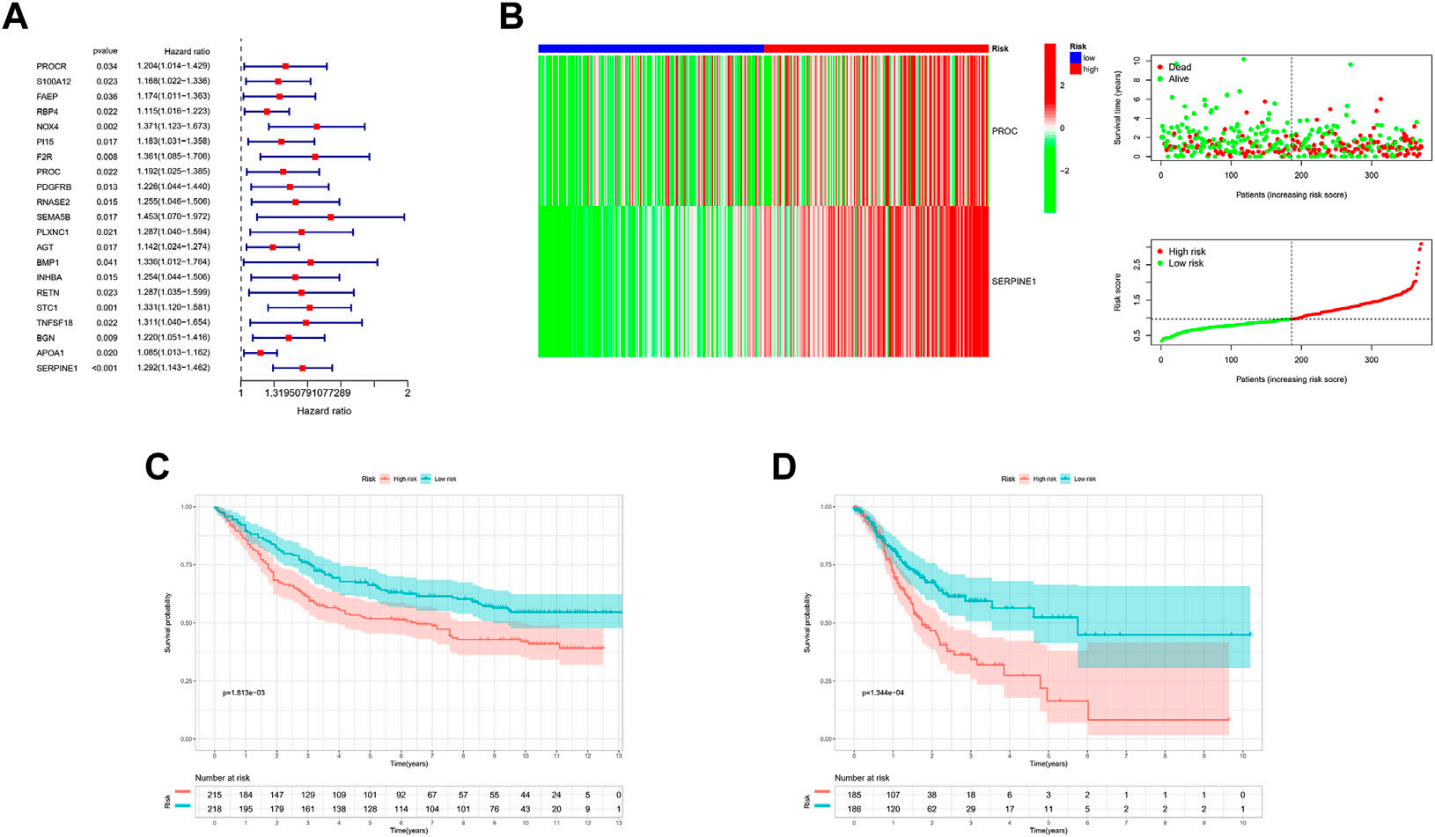
Establishment Risk Assessment Model and Survival outcomes in GC. **(A)** 21 CD4+ T cells-related hub genes Through a univariate Cox survival analysis in MEblue module. **(B)** The distribution plot of the Risk score with survival times. **(C, D)** Kaplan-Meier analysis of the OS between the two risk groups in the GSE84437 and TCGA cohort.

Through a univariate Cox survival analysis, 21 CD4^+^ T cell-related hub genes among the 173 genes in MEblue module deemed closely correlated with GC patient are set for follow-up analysis, as shown in Figure 4A (*p* < 0.05, log-rank test). To determine the best independent prognostic genes, lasso and multivariate Cox regression analysis for OS was performed among those 21 CD4^+^ T cell-related hub genes (Supplementary Figure S3). There were only 2 genes (PROC, SERPINE1) that significantly affected the OS of GC patients. Meanwhile, based on the selected CD4+T cells-related hub genes, we constructed a prognostic index for all cancer samples. The risk model was established by multiplying expression data of hub genes by the Cox regression coefficient, such as follows: risk-score = expression level of PROC*(0.193,582) + expression level of SERPINE1*(0.267008). The distribution plot of the risk score demonstrated that the survival times were reduced while the risk-score increased in Figure 4B.

Finally, we used the identified cut-off point to re-distinguish high-risk groups from low-risk groups in the cohort for validation. As illustrated in Figures 4C,D, the patient within low-risk groups had a better OS than the high-risk patients (*p* < 0.05, log-rank test) no matter the TCGA cohort or GEO cohort.

### The 2 gene survival and GSEA of risk model in GC

**FIGURE 5:**
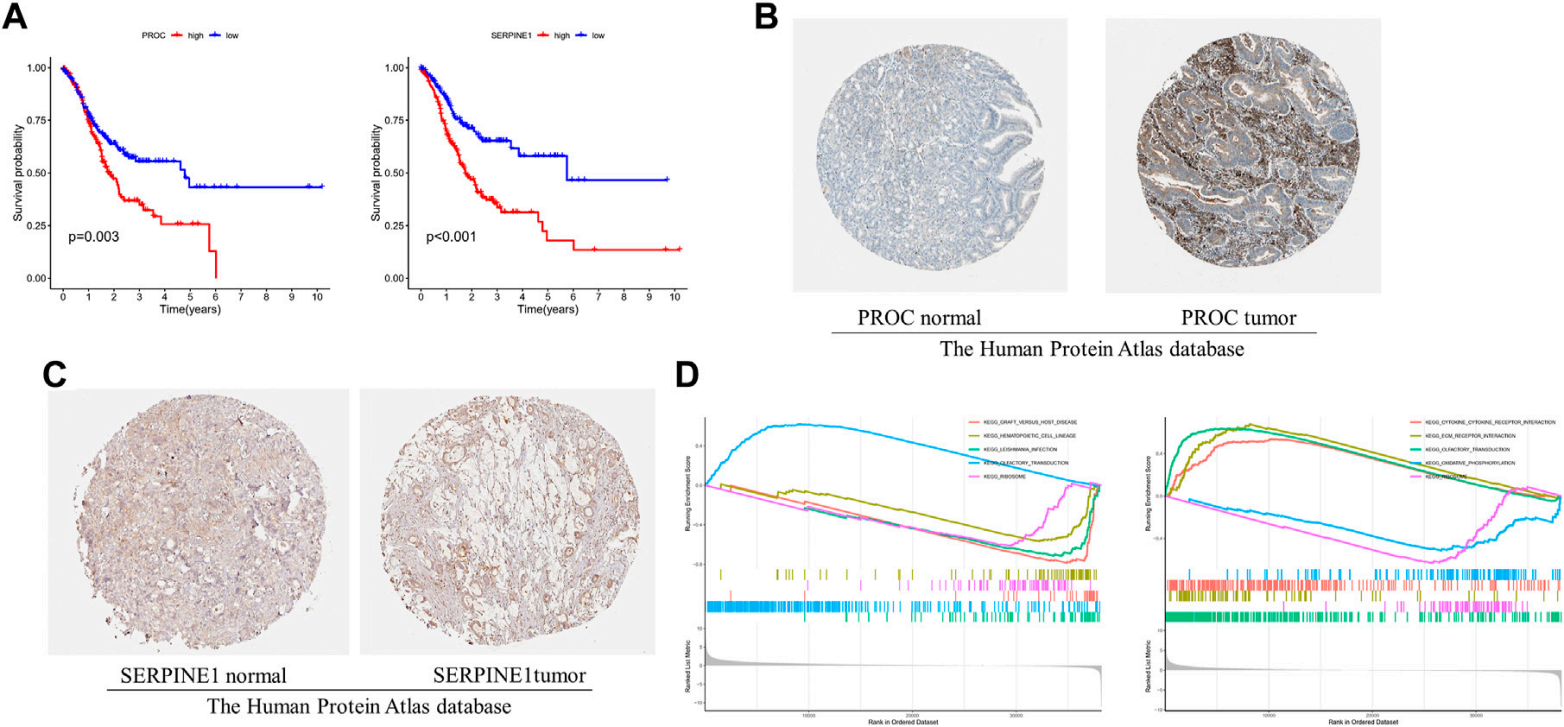
The 2 gcnc survival and GSEA of risk model in GC. **(A)** The relationship between gene expression of PROC and SERPINE I and OS. **(B, C)** Protein expression of PROC and SERPINEI in tumor and normal GC tissue. **(D)** The top 5 GSEA terms of PROC and SERPINEI. .

The GC patient with low-expression of PROC and SERPINE1 had a better OS than the high-expression patients (Figures 5A,B). Consistent with our results, compared to normal tissues, the protein expression of PROC and SERPINE1 were both significantly higher in GC tissues (Figures 5C,D). In addition, GSEA was conducted to identify gene sets associated with the different risk subgroups. The top 5 GSEA terms of PROC and SERPINE1 were illustrated in Figures 5E,F, respectively. The genes of PROC were enriched in graft *versus* host disease, hematopoietic cell lineage, leishmania infection, olfactory transduction, and ribosome. While, the gene of SERPINE1 were enriched in aminoacyl-tRNA biosynthesis, cell cycle, DNA replication, pyrimidine metabolism, and spliceosome. Detailed GSEA results can be viewed in Supplementary Table S3.

### *In vitro* validation and survival analyses

**FIGURE 6:**
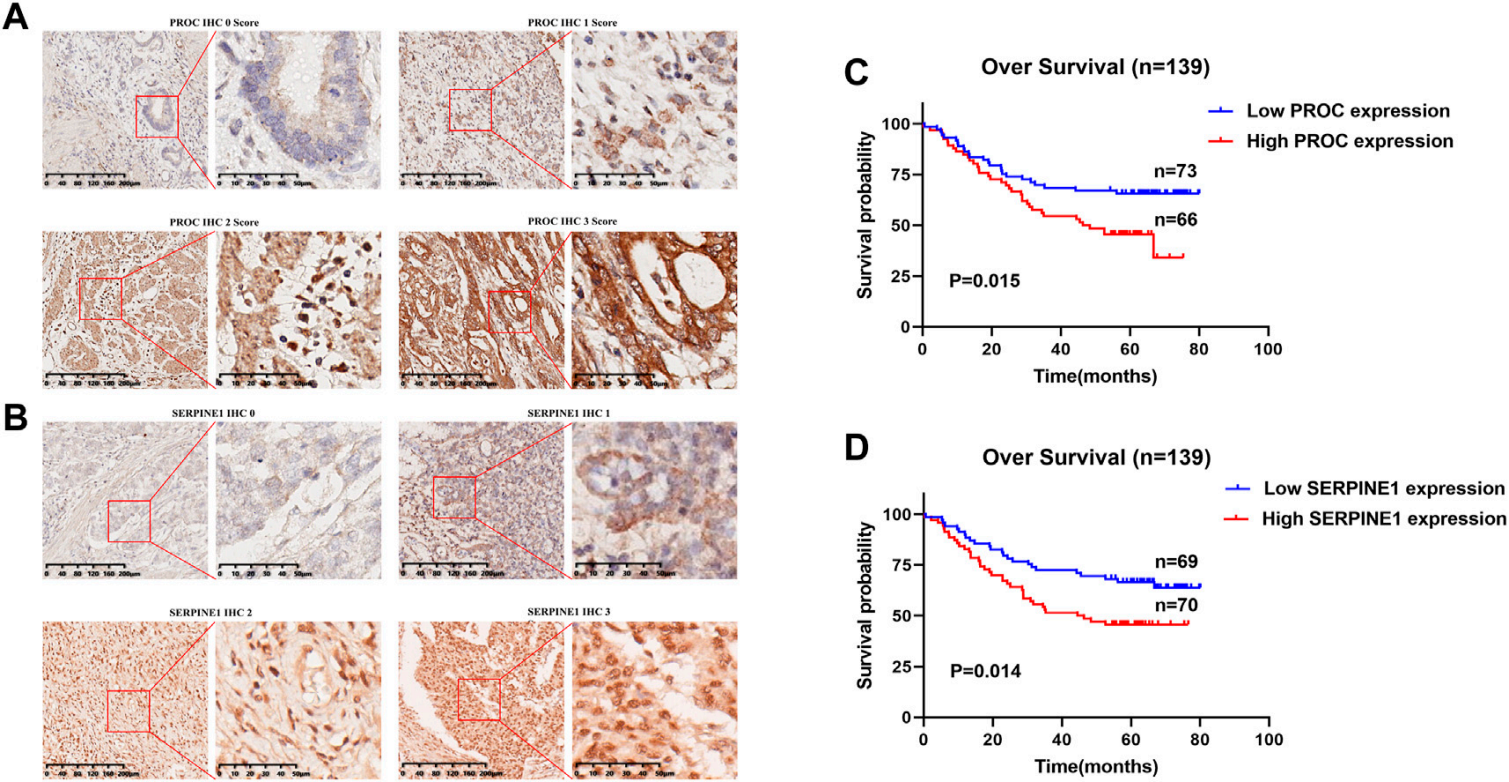
In Vitro Validation and Survival Analyses. **(A)** IHC staining of the PROC protein in GC tissues. **(B)** IHC staining of the SERPINEI protein in GC tissues. **(C)** Survival analyses of the PROC protein in GC patients. **(D)** Survival analyses of the SERPINEI protein in GC paticnts.

PROC and SERPINE1 genes were further validated *in vitro*. Immunohistochemistry and western blotting experiments on 139 paired GC patients and normal tissue samples showed that the protein expression of PROC and SERPINE1 were significantly higher in GC samples than in normal tissue samples (Figures 6A,B). Moreover, patients with high expression of PROC and SERPINE1 were found to have worse prognosis in our cohort (Figures 6C,D). These findings were consistent with the results obtained from the GC cohort of TCGA.

### Construction of a nomogram to assess survival

**FIGURE 7:**
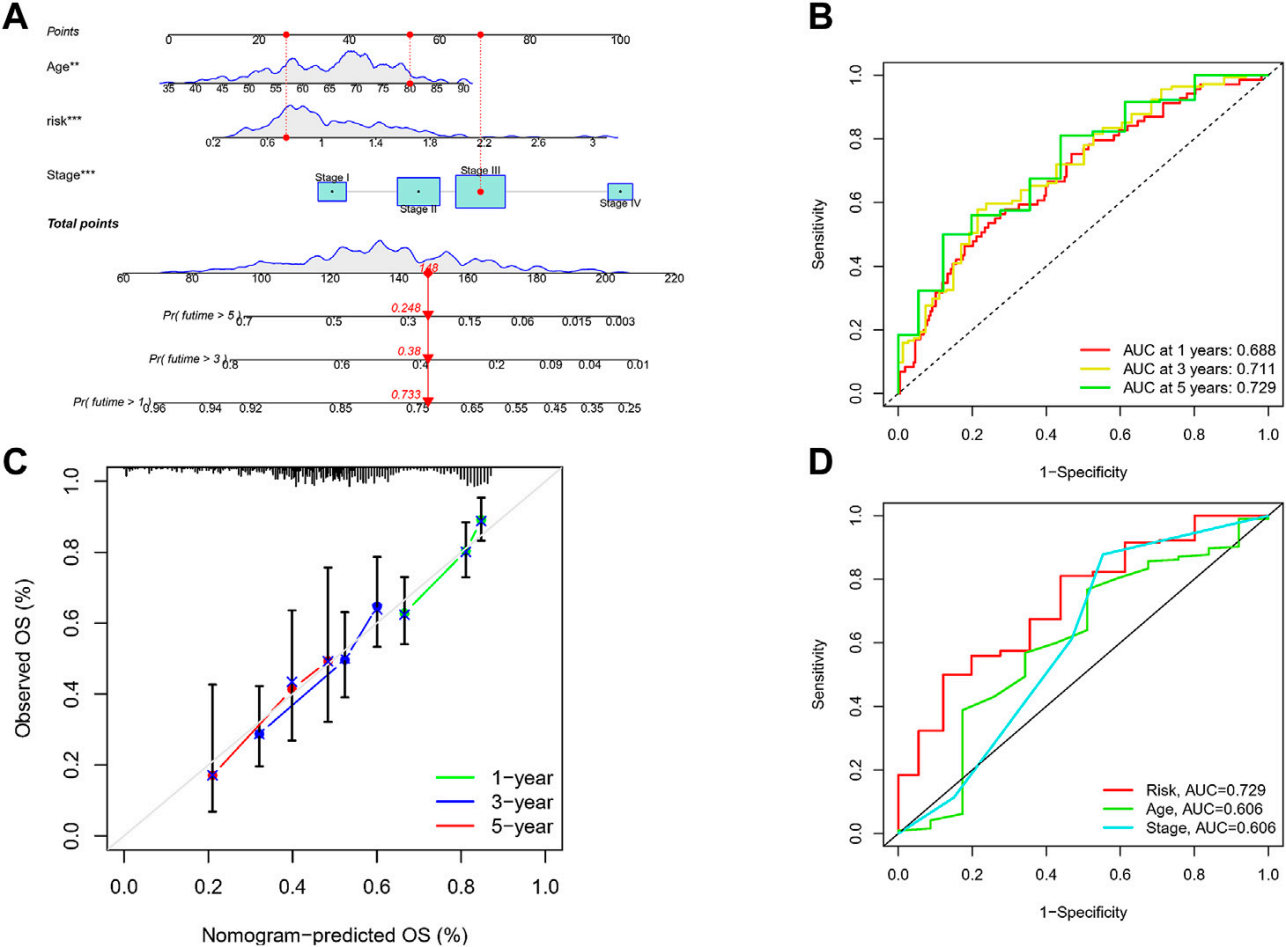
Construction of a nomogram to assess survival. **(A)** Nomogram for prcdicting the 1-, 3-, and 5-year OS of GC patients in TCGA cohort. **(B)** ROC curves for predicting the I-, 3-, and 5-year ROC curves in TCGA. **(C)** Calibration curves of the nomogram for predicting of I-, 3-, and 5-year OS in the TCGA. **(D)** The prediction accuracy of nomogram and the TNM stage in the TCGA.

Given the inconvenient clinical value of the risk-score in predicting OS in patients with GC, a nomogram incorporating risk-score and clinicopathological characteristics was developed to predict 1-, 3-, and 5-year OS rates in patients with GC (Figure 7A). For TCGA, our AUC studies on the nomogram model revealed a good accuracy for OS at 1-, 3-, and 5- years (Figure 7B). In TCGA, the proposed nomogram performed similarly to an ideal model according to the calibration plots (Figure 7C). Finally, we compared the nomogram’s prediction accuracy to that of the TNM stage in the TCGA (Figure 7D). The results illustrated that the nomogram’s AUC values were greater than the TNM stage in three cohorts.

### Characteristics in clinicopathology and gene mutation in GC

**FIGURE 8:**
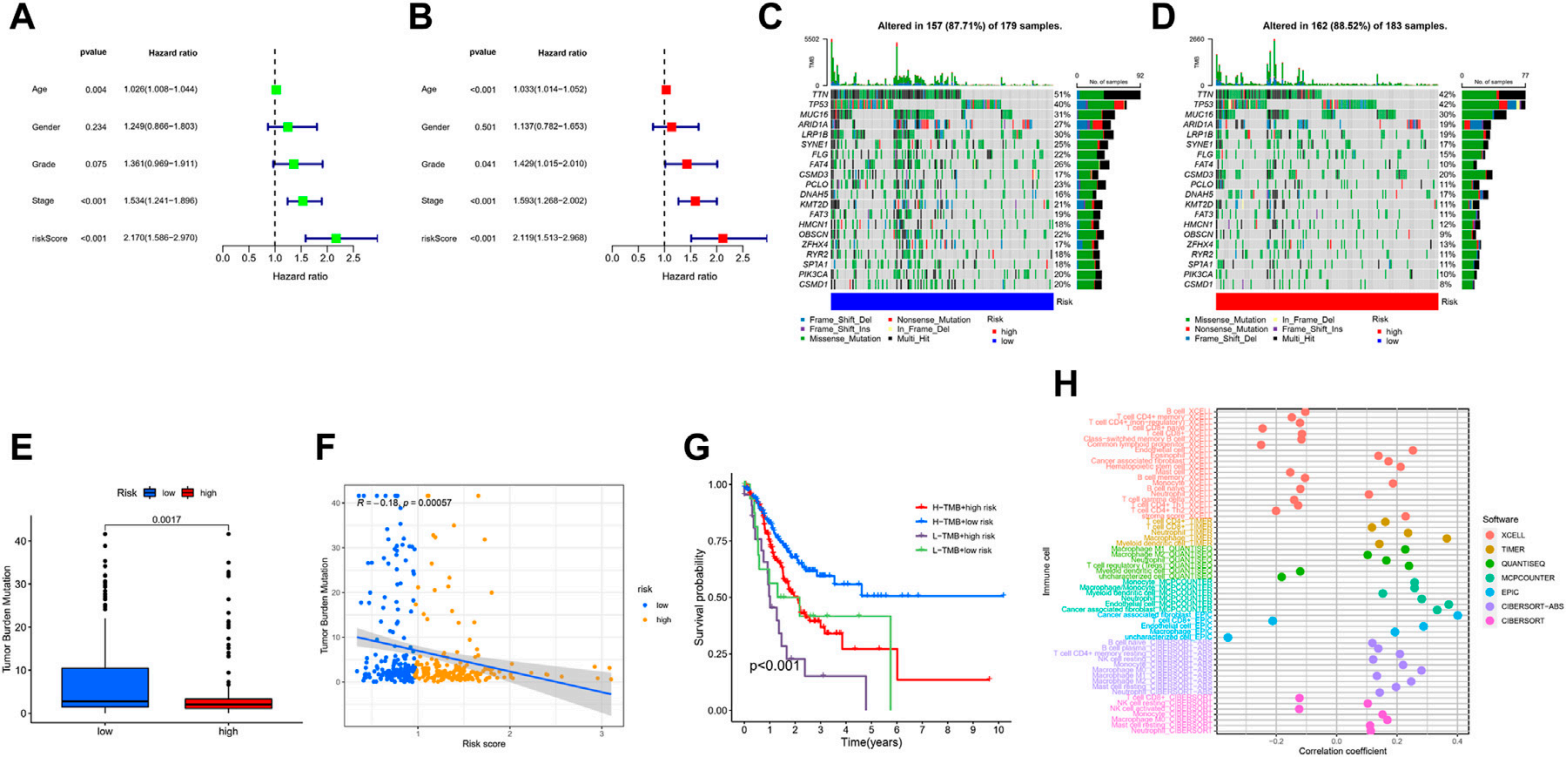
Characteristics in clinicopathology and gene mutation in GC. **(A, B)** The univariate and multivariate cox regression analysis in Risk-score subgroups. **(C, D)** Significantly mutated genes in the mutated GC samples of the high and the low risk groups, respectively. Mutated genes (rows, top 20) are ordered by mutation rate; samples (columns) are arranged to emphasize mutual exclusivity among mutations. The right shows mutation percentage, and the top shows the overall number of mutations. The color-coding indicates the mutation type. **(E)** The TMB of two different risk subgroups. **(F)** Relationships between Risk-score and TMB. **(G)** The prognosis of GC in different Risk-score and TMB subgroup. **(H)** The prognosis of GC in different Risk-score and TMB.

Univariate Cox regression analysis illustrated that risk-score and stage were significantly associated with the prognosis of GC (Figure 8A). Multivariate Cox regression analysis showed that risk-score is an independent prognostic factor after adjusting for other clinicopathologic factors (Figure 8B). We analyzed the gene mutations to further understand the immunological nature in different risk subgroups. We identified the top 20 genes with the highest mutation rates in the high-risk subgroup (Figure 8C) and low-risk subgroup (Figure 8D). The results showed that missense mutation was the most common mutation type. The mutation rates of TTN, TP53, and MUC16 were not only higher than 25% in both groups, but are also the most common in both groups. Additionally, we analyzed the relationship between the risk-score and tumor mutational burden (TMB). The expression of TMB was significantly higher in the low-risk subgroup than in the high-risk subgroup (Figure 8E). Moreover, risk-score was correlated with TMB in gene subtypes (r = −0.18, *p* < 0.05), as revealed in Figure 8F. Finally, we found that high TMB was associated with longer survival time, with the effect of higher TMB on prognosis more obvious in the low-risk group. This may be related to the immune cells in the tumor microenvironment (Figures 8G,H).

### Drug sensitivity

**FIGURE 9:**
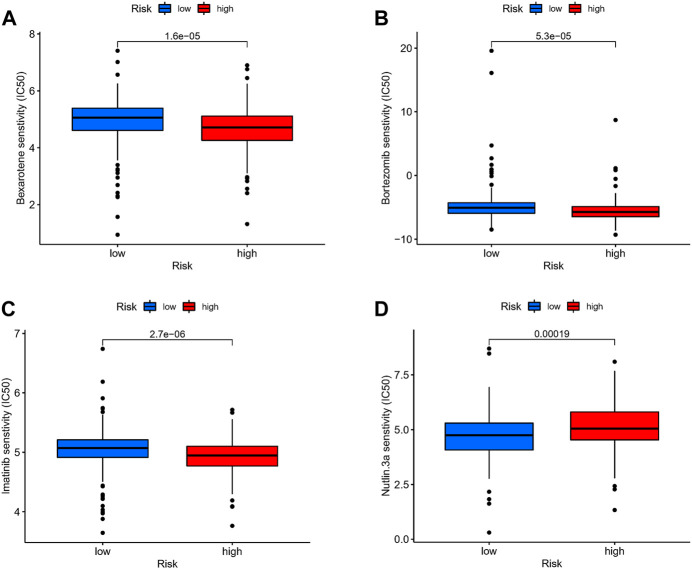
Drug sensitivity. **(A–D)** The relationship between different risk groups and the effectiveness of chemotherapy for treating GC in the TCGA cohort.

We attempted to discover the relationship between different risk groups and the effectiveness of chemotherapy for treating GC in the TCGA cohort. We elucidated that low risk was associated with a lower half maximal inhibitory concentration (IC50) of chemo-therapeutics such as Nutlin.3a (*p* < 0.05), whereas high risk was associated with a lower IC50 in drugs such as Bexarotene, Imatinib, and Bortezomib (*p* < 0.05). Therefore, Figures 9A–D revealed that Risk-score acted as a potential predictor for chemo-sensitivity and the detail can be found in Supplementary Figure S4.

### Relationship of risk-score with immune checkpoint inhibitors and immunotherapy prediction

**FIGURE 10:**
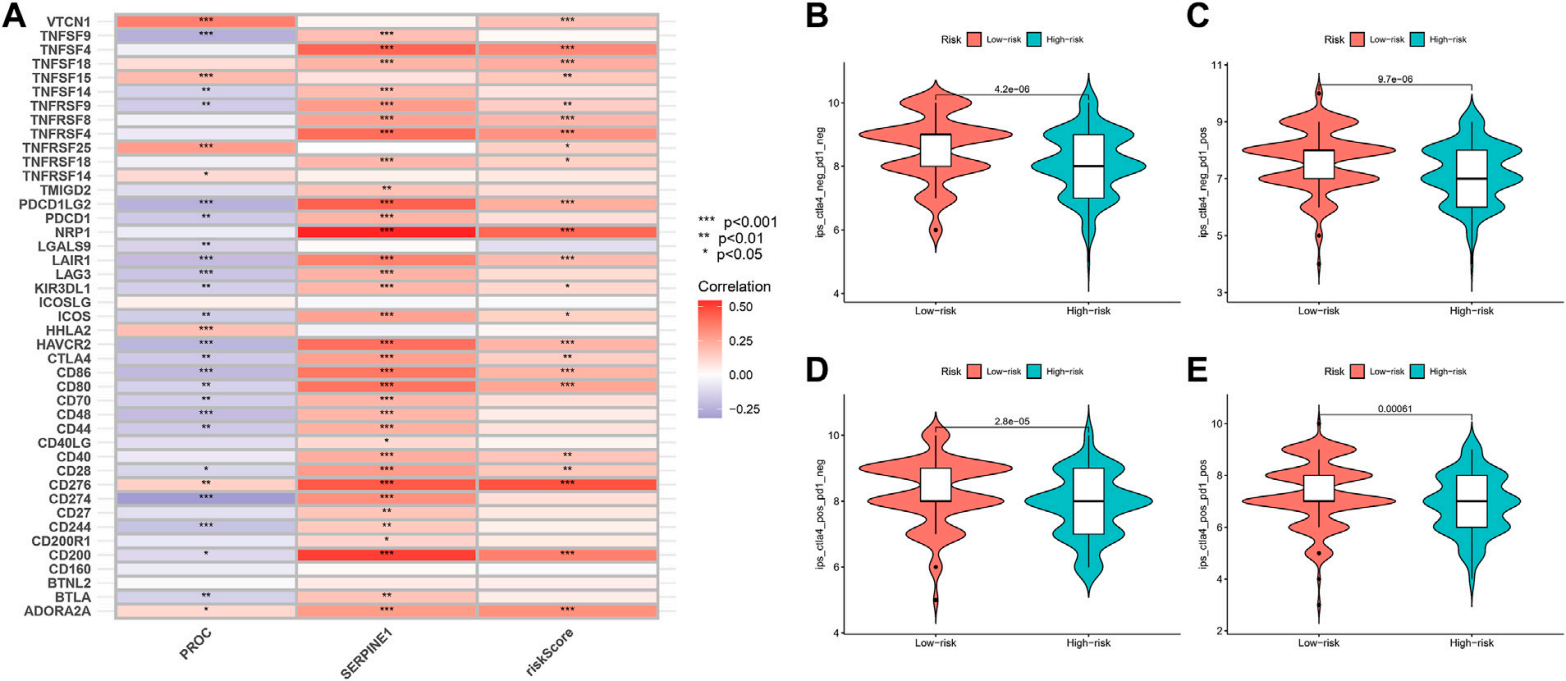
Relationship of Risk-Score with Immune checkpoint inhibitors and Immunotherapy prediction. **(A)** The relationship between PROC, SEP,PINEI, Risk-score and immune checkpoints. **(B–E)** The vioplot of the difference expression of Cf LA4 and PD- I between high- and low-risk groups.

In our study, we found an inverse relationship between the number of CD8^+^ T cells and the degree of risk in the model (Supplementary Figure S5). In addition, we found that these two genes and risk-score are highly related to most immune checkpoints, such as CTLA4 and CD274 (PDL-1) (Figure 10A). As such, it is no surprise that immune checkpoint inhibitors have achieved good clinical results in recent years, even replacing and becoming the main treatment in some malignant tumors.

In order to assess the potential efficacy of clinical immunotherapy in different risk subgroups, we analyzed the correlation between the risk-score and IPS in GC patients to predict the response of ICIs. For IPS, the immune checkpoints were CTLA-4, PD-1 and PD-L1. Therefore, their immune checkpoints were utilized to evaluate the potential of ICI treatment (Figures 10B–E). As a result, we found that the immune response was significantly elevated in the low-risk group, which means that the ICIs will illicit more immunogenicity in the low-risk group. Collectively, these results suggested that the low-risk group will benefit more from immunotherapy due to a better immune response.

## Discussion

GC is one of the human malignant tumors with the highest incidence in the world, but because the early symptoms are not obvious, most patients are diagnosed late stage (Nie et al., 2020), and the recurrence rate in these later stages are high, with poor prognosis and high rates of fatality (Galon and Bruni, 2020). In recent years, due to the incredibly complex and heterogeneous tumor microenvironment, more and more attention has been paid to the changes of tumor-associated immune cells. Many reports have pointed out that evading immune surveillance is one of the bases for the occurrence and development of cancer (Griss et al., 2019). Studies have concluded that the type and proportion of immune cell infiltration are closely related to the clinical outcome of patients (Zeng et al., 2019). IRGs participate in the regulation of the immune system and play an important role in the complex regulatory network of tumors. Thus, as our understanding grew, immunotherapy has sprouted as a new treatment method, and is now actively applied to a variety of tumors, with its efficacy verified (Das et al., 2020).

Therefore, in this study, we first analyzed the differentially expressed genes between gastric cancer samples and normal tissues in the TCGA database, and found hundreds of immune-related genes that may be meaningful. At the same time, we analyzed the types of immune cells in GC and explained the close correlation between them through CIBERSORT. Then, we selected the key modules from among them through WGCNA. Our study found that MEblue is a key module of gastric cancer, which contains gene groups related to the level of activated CD4 memory T Cell infiltration. Currently, it is known that CD4^+^ T cells have a variety of ways to kill tumor cells, and memory T cells are a subset of CD4^+^ T cells. However, there are few studies on this kind of cells at present, so exploring the function of activated CD4^+^ T cells has important research significance. Studies have shown that CD4 memory T cells are associated with better survival in patients with gastric cancer. Such as, Activin-A can enhance the anti-tumor ability of the body by preventing the depletion of CD4 T cells (Morianos et al., 2021). In mice, IL11 can inhibit the anti-tumor effect mediated by CD4^+^ T cells (Huynh et al., 2021). Activated CD4 T cells can enhance antigen reactivity through NOTCH signaling pathway (Wilkens et al., 2022). GO and KEGG pathway enrichment analysis found that many of the above genes are related to leukocyte chemotaxis and neutrophil migration signal pathways, and these components play an important role in tumor microenvironment and affect tumor occurrence and development. According to study, MMP-9 is mainly secreted by neutrophils. By up-regulating the expression of MMP-9, SETDB1 can promote the occurrence and metastasis of gastric cancer (Shang et al., 2021). IL-17 can promote the proliferation and migration of gastric cancer cells by targeting SLP1 (Xu et al., 2020). CXCL5 can promote the metastasis of gastric cancer by inducing epithelial-mesenchymal transformation and activating neutrophils (Mao et al., 2020). Based on the regression analysis of the above genes, we found that only two genes (PROC and SERPINE1) had significant effects on OS in patients with gastric cancer.

SERPINE1 is an important inhibitor of serine protease and plays a major role in signal transduction, cell adhesion and cell migration (Simone et al., 2014; Zhang Q. et al., 2020). Plasminogen activator inhibitor-1 (PAI-1), the coding product of SERPINE1 gene, is a key regulator of tissue plasminogen activator (tPA) and urokinase plasminogen activator (uPA), and is a member of the plasminogen activator system. As a secretory protein, increased activity of PAI-1 increases the risk of metastasis in melanoma (Hanekom et al., 2002). In patients with breast cancer, elevated plasma PAI-1 level is a potential prognostic marker as well (Palmirotta et al., 2009; Ferroni et al., 2014). In GC, LncRNA NKX2-1-AS1 promotes tumor progression by up-regulating the expression of SERPINE1. SERPINE1 is an effective biomarker related to epithelial mesenchymal transformation of GC (Xu et al., 2019). In addition, the expression of PAI-1 is abnormal in ovarian cancer, glioma, renal clear cell carcinoma and other tissues, and is related to the poor prognosis of patients (Liao et al., 2018; Peng et al., 2019; Vachher et al., 2020; Ahluwalia et al., 2021; Ma et al., 2021; Zhao et al., 2021). Furthermore, as a protein encoded by the PROC gene, protein C is known to engage in hemostasis, inflammation, and signal transduction, and it has a protective effect on the endothelial barrier as well. In recent years, protein C has been recognized for its importance in a multitude of diseases, including sepsis, myocardial infarction, and cancer (Griffin et al., 2015). For example, activated protein C cross-activates sphingosine-1-phosphate receptor-1 (S1P1) in cancer thus leading to greater cell-to-cell junction stability, thereby decreasing extravasation (Van Sluis et al., 2009). In fact, addition of activated protein C *in vitro* decreases endothelial adhesion and transmigration of melanoma cells (Bezuhly et al., 2009). Other researches show that activated PROC-PROCR-F2R axis can stimulate the MAPK pathway via activation of epidermal growth factor receptor (EGFR) to promote the progression of breast cancer (Gramling et al., 2010). Additionally, activated protein C promotes anticoagulation of cancer cell microenvironment and upregulates cancer cell migration in ovarian cancer (Althawadi et al., 2015). However, at present, there is not much research on PROC gene in gastric cancer, so we predict that this gene may be associated with gastric cancer.

Our study found that age, risk-score and pathological stage were significantly associated with the prognosis of gastric cancer. Gastric cancer is an age-related disease because the overall survival outcome of elderly cancer patients is poor (Nelen et al., 2018). In the latest edition of AJCC, In et al. found that pathological stage was closely related to the prognosis of gastric cancer (In et al., 2017). Therefore, in order to better improve the accuracy of prognosis prediction, we combined risk-score with age and pathological stage to construct a line map to predict the OS of patients with gastric cancer. The results show that the line chart has high accuracy in OS prediction and is close to the ideal model after correction. And in the test of TCGA data set, the prediction accuracy of line chart risk-score is better than that of TMN stages. The results showed that risk-score was an independent prognostic factor. Chemotherapy is one of the important treatment methods for patients with gastric cancer, which generally needs to be evaluated after several cycles of treatment, so as to judge the efficacy of those chemotherapeutic drugs. In the calculation, we found that there are great differences in drug sensitivity among different risk groups, which may help patients with gastric cancer to select sensitive drugs before chemotherapy and reduce potential trial and error, potentially prolonging survival time.

A large number of studies on various tumors have shown that patients with high TMB tend to have a good survival rate (Cheng et al., 2022). Similarly, our study found that higher TMB was found in patients with low risk-score. The OS of patients with high TMB was significantly longer than that of patients with low TMB. In some literatures, MUC16 mutation is associated with better prognosis and higher TMB in gastric cancer, while TTN mutation is associated with immune checkpoint blockade in solid tumors (Li et al., 2018; Yang et al., 2020). Although TP53 is one of the most common mutant genes, it is not enough to correctly predict the prognosis of patients (Li L. et al., 2020; Wang et al., 2022). Therefore, gastric cancer patients with low risk-score benefit better from immunotherapy. Our study found that risk-score is closely related to many immune cells, such as macrophages, dendritic cells, NK cells, B Cells, T cells and so on, and these cells play an important role in the tumor microenvironment. For example, CD80 and CD86 are markers of M1 macrophages, which can inhibit tumor growth of gastric cancer (Xie et al., 2020). CD40 is a marker of dendritic cells. Its main roles in anti-tumor immune response are phagocytosis of dead tumor cells, capture and presentation of tumor-associated antigens and activation of various T cells, thus stimulating a series of immune responses to kill tumor cells (Murphy and Murphy, 2022). CD48 is a marker of NK cells. Inhibiting the function of NK cells can promote the growth of gastric cancer cells (Guo et al., 2021). Therefore, the TME score can reflect the prognosis of the tumor.

It has been reported that CD8^+^ T cells are a highly destructive immune effector cell population in anti-tumor response. After activation, they form CD8^+^ cytotoxic T cells, which circulate to the tumor site to induce immune clearance (Farhood et al., 2019). Immune checkpoint blockade therapy uses immune checkpoint blocking agents to relieve the inhibitory pressure on CD8^+^ T cells and restore their sensitivity and killing ability to tumor cells (Darvin et al., 2018). In our study, it was found that CD8^+^ T Cell infiltration in the low-risk group was significantly higher than that in the high-risk group and had a stronger killing effect on the tumor. To find out which gastric cancer patients are sensitive to immunotherapy, we investigated the sensitivity of two risk subgroups to ICI. In our study, we used IPS to explore risk-score based on TME differences that may reflect the different immune benefits of ICI therapy. IPS is mainly associated with several immune checkpoints, including CTLA4, PD-1 and PD-L1. Our study shows that the low-risk group has a higher potential for ICI response. For clinical trials of immunotherapy, the literature shows that pd-1 has anti-tumor activity and is safe for patients with GC. It has been included in NCNN guidelines as an important treatment for advanced gastric cancer (Ajani et al., 2022). Consistent with our results, mortality was significantly lower in the low-risk group (classified according to Risk-score score). Based on the results of IPS, we found that risk-score can distinguish the different outcomes of individuals receiving immunotherapy. Risk-score as a predictive score is expected to provide a theoretical basis for the selection of ICI treatment in clinical trials. This means that further research can focus on the combination therapy of patients with gastric cancer, and the predictive model may help to accelerate the development of personalized cancer treatment.

In conclusion, our research shows that risk-score plays a very important role in analyzing the clinicopathological features, immune infiltration and clinical prognosis of patients with gastric cancer. In addition, this study also revealed the role of risk-score in predicting the prognosis, and provided a guide to immunotherapy combined with chemotherapy in GC patients. However, the interaction between these model genes and their biological mechanisms needs to be further studied.

## Data Availability

The original contributions presented in the study are included in the article/Supplementary Material, further inquiries can be directed to the corresponding authors.
