# Supplementary material for: The predictive effect of immune therapy and chemotherapy under T cell-related gene prognostic index for Gastric cancer
Source: Front Cell Dev Biol. 2023 May 18;11:1161778. doi: 10.3389/fcell.2023.1161778 (PMC10232754; doi:10.3389/fcell.2023.1161778)
Supplement: Supplementary file 1 [file DataSheet1.zip › Supplementary Material/Supplementary Image 3.PDF]

Supplementary Figures

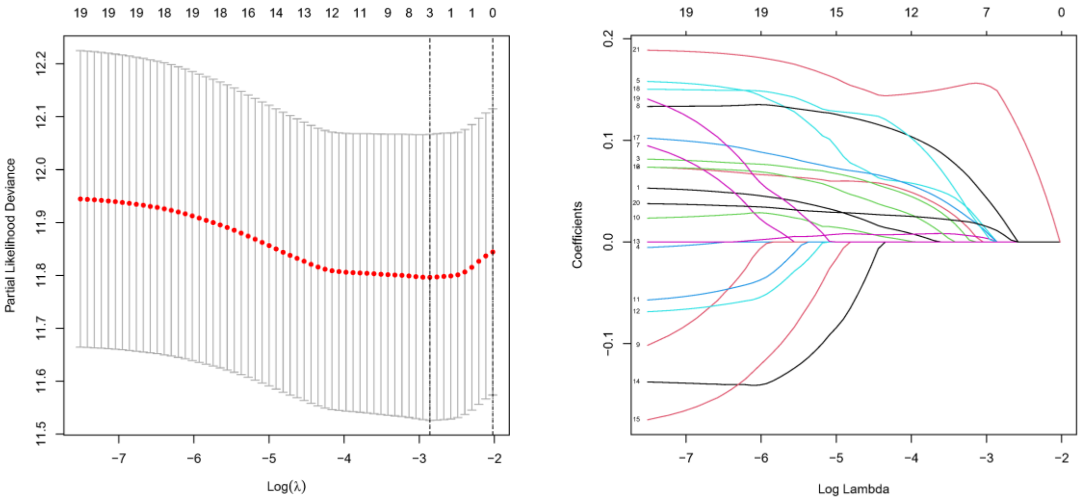

Supplementary Figure S3: Lasso Cox analysis for OS of the 21 CD4+ T cells-related hub genes.
