# Supplementary figures and images for: The predictive effect of immune therapy and chemotherapy under T cell-related gene prognostic index for Gastric cancer
Source: Front Cell Dev Biol. 2023 May 18;11:1161778. doi: 10.3389/fcell.2023.1161778 (PMC10232754; doi:10.3389/fcell.2023.1161778)

## Supplementary Figures

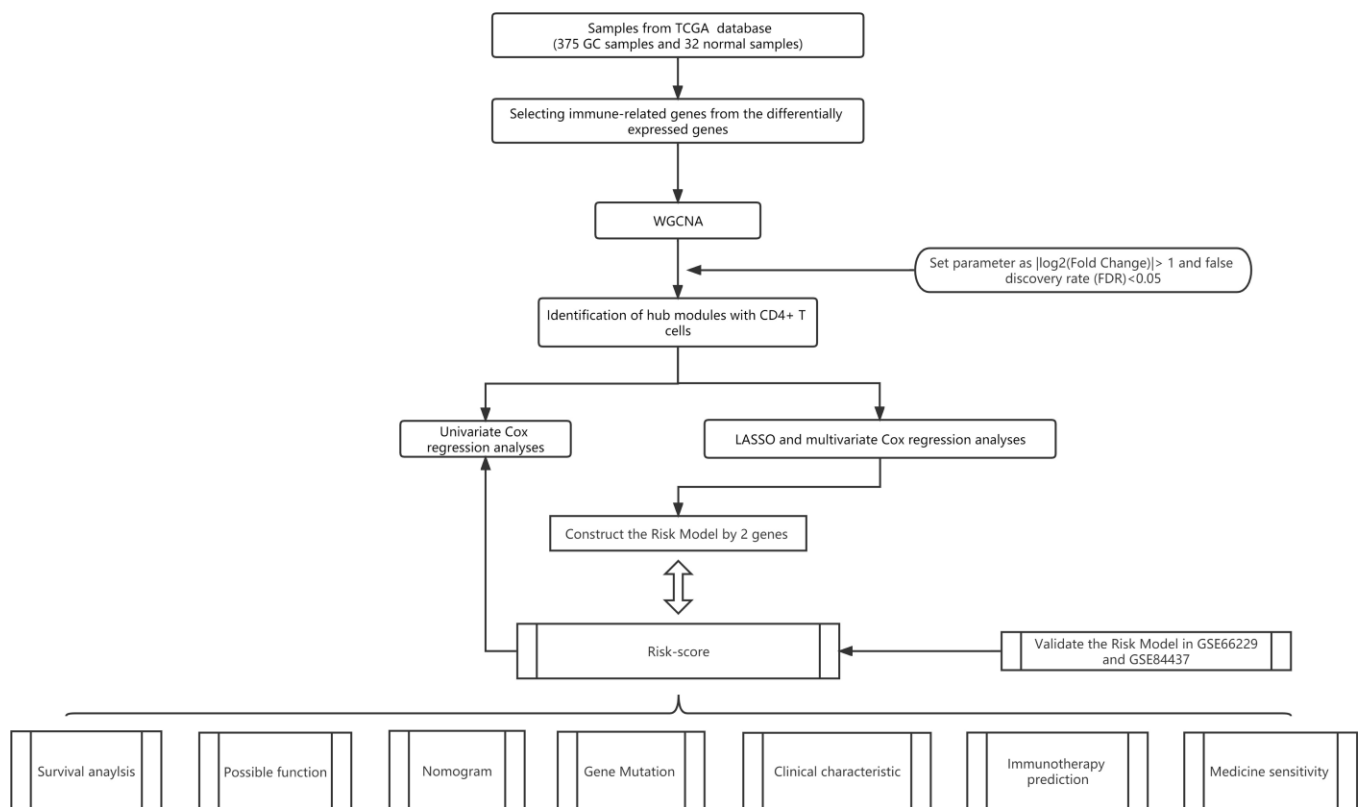

Supplementary Figure S1. The flow chat of the study.

Supplement: Supplementary file 1 [file DataSheet1.zip › Supplementary Material/Supplementary Image 1.PDF]
